# COVID-19 in Dental Settings: Novel Risk Assessment Approach †

**DOI:** 10.3390/ijerph18116093

**Published:** 2021-06-05

**Authors:** Ali Alsaegh, Elena Belova, Yuriy Vasil’ev, Nadezhda Zabroda, Lyudmila Severova, Margarita Timofeeva, Denis Dobrokhotov, Alevtina Leonova, Oleg Mitrokhin

**Affiliations:** 1 Institute of Dentistry Named after E.V. Borovsky, I.M. Sechenov First Moscow State Medical University (Sechenov University), Trubetskaya St. bldg. 8\2, 119435 Moscow, Russia; 2 Department of General Hygiene, I.M. Sechenov First Moscow State Medical University (Sechenov University), Trubetskaya St. bldg. 8\2, 119435 Moscow, Russia; ms.ekochina@mail.ru (E.B.); zabroda_n_n@staff.sechenov.ru (N.Z.); alichka-812@mail.ru (A.L.); mov1163@yandex.ru (O.M.); 3 Department of Operative Surgery and Topographic Anatomy, I.M. Sechenov First Moscow State Medical University (Sechenov University), Trubetskaya St. bldg. 8\2, 119435 Moscow, Russia; dr.vasiliev@gmail.com; 4 Department of Phthisiopulmonology and Thoracic Surgery named after M.I. Perelman, I.M. Sechenov First Moscow State Medical University (Sechenov University), Trubetskaya St. bldg. 8\2, 119435 Moscow, Russia; lyudmila.severova.1992@mail.ru; 5 Department of Medical Law, I.M. Sechenov First Moscow State Medical University (Sechenov University), Trubetskaya St. bldg. 8\2, 119435 Moscow, Russia; timofeevainfo@mail.ru; 6 Department of Chemistry, I.M. Sechenov First Moscow State Medical University (Sechenov University), Trubetskaya St. bldg. 8\2, 119435 Moscow, Russia; dennicas@mail.ru

**Keywords:** COVID-19, SARS-CoV-2, risk assessment, dental care, oral health, dentistry, infection control, occupational safety, public health, hygiene

## Abstract

The novel coronavirus (COVID-19) outbreak is a public health emergency of international concern, and this emergency led to postponing elective dental care procedures. The postponing aimed to protect the public from an unknown risk caused by COVID-19. At the beginning of the outbreak, for public health authorities, the aerosol-generating procedures and the close proximity between dental care workers and patients in dentistry represented sufficient justification for the delay of dental visits. Dental care is a priority, and for many years, studies have proven that the lack and delay of dental care can cause severe consequences for the oral health of the general population, which can cause a high global burden of oral diseases. Safety is necessary while resuming dental activities, and risk assessment is an efficient method for understanding and preventing the COVID-19 infectious threats facing the dental industry and affecting dental care workers and patients. In this study, for safe dental care delivery, we adapted risk assessment criteria and an approach and an occupational classification system. Based on those tools, we also recommend measures that can help to minimize infectious risk in dental settings.

## 1. Introduction

The novel coronavirus (COVID-19) outbreak that emerged in December 2019 became an international health issue. Records of the Center for Systems Science and Engineering, Johns Hopkins University (April 2021) showed over 149,000,000 COVID-19 registered cases with over 3,000,000 global deaths. The data also showed that internationally, the United States (US) is the most affected country, followed by India, Brazil, France, Russia, Turkey, and the United Kingdom (UK) [1,2].

COVID-19 led to a global healthcare crisis, and dental care workers among all healthcare providers are most affected. Interestingly, many reports highlighted the high infectious risk of dental care delivery because of the proximity to the oral cavity. In 2020, as a response to this risk, many dental public health authorities, including the American Dental Association (ADA) and World Health Organization (WHO), called for postponing elective dental procedures at the beginning of the pandemic. Those decisions seemed to be reasonable to protect public health [3,4,5,6,7,8].

Later, in the United States (US), a study published in the November issue of the Journal of the American Dental Association (2020) found that fewer than 1% of dentists nationwide were estimated to be COVID-19 positive. The ADA respectfully disagreed with the decisions of WHO and based on this study, ADA resumed dental care delivery and recommended using the highest level of personal protective equipment available such as masks, goggles, and face shields. To control the risk of generated aerosols, ADA also recommended the usage of rubber dams and high-velocity suction, and hand scaling instead of ultrasonic scaling when cleaning teeth [9,10,11].

In a survey of dental care providers working for 600 practices in the United Kingdom (UK), 96% stated there is a need for greater access to affordable dental care because lockdown has an adverse impact on oral health. 88% percent of dental experts surveyed said that dental health in the UK could decline because a lack of routine appointments could lead to preventable dental issues getting worse [12].

According to the WHO, oral health is an indicator of overall health, well-being, and quality of life. Postponing dental procedures can have significant consequences for the oral health of the population. This delay in receiving dental care can cause caries lesions and periodontitis, which eventually can increase the possibility of dental pain or tooth loss. This issue is crucial since the COVID-19 pandemic can markedly affect oral health. A study on the global burden of oral conditions that oral health problems accounted for 15 million disability-adjusted life years, implying an average health loss of 224 years per 100,000 population [13,14].

It is necessary to highlight the negative economic aspect of delayed dental care; late dental treatment can cause higher treatment expenses. Studies on global economic impact of dental diseases estimated that internationally, direct treatment costs due to dental diseases at 298 billion USD per annum, corresponding to an average of 4.6% of global health expenditure. Indirect costs amounted to 144 billion USD per annum, corresponding to economic losses within the range of the ten most frequent global causes of death. Within the limitations of currently available data sources and methodologies, these findings suggest that the global economic impact of dental diseases amounted to 442 billion USD in 2010. Postponing dental visits during the pandemic can cause damages to the global economy, and promoting oral health may imply substantial economic benefits to reduce treatment costs and cause fewer productivity losses in the labor market [15].

Internationally, the dental industry and oral health are already facing enough non-COVID-19 related issues resulting in delays of dental care delivery, including negligence or “wait and see” behavior, poor dental services, visits with no treatments, financial reasons, lack of time, and dental fear. According to the Global Burden of Disease Report of WHO (2017), oral diseases affect close to 3.5 billion people worldwide, with caries of permanent teeth being the most common condition. About 2.3 billion people suffer from untreated dental caries, while 530 million children have caries. These statistics show that dental caries one of the most common global public health concerns, and delaying dental care delivery is not a wise decision [16,17].

During the pandemic, an indicator of the consequences of delaying dental care is an increase in the number of reports regarding that coronavirus infection has a possibility of causing a direct negative effect on the oral health of the population because of various consequences such as xerostomia, therapeutic measures, and other complications [18].

A published scoping study with a systematic search approach showed that dental health care workers have many COVID-19 related concerns, including economic, ethical, social, and professional factors. Resolution of those concerns may involve enhancing coping strategies relating to patient management and infection control strategies and using new technologies for virtual contact with the patient with no risk of infection [19].

Dental care is a priority, and studies proved the possibility of safely administering dental care even in high-risk patients. Assessing the infectious risk is essential for the safety of dental personnel and patients. The primary purpose of risk assessments is to identify health and safety hazards, evaluate the risks presented within the workplace, and evaluate the effectiveness and suitability of existing control measures [20,21].

A multidisciplinary approach is essential in developing risk assessment approaches and precautionary guidelines. The FDI World Dental Federation (the global voice of the dental profession that represents over one million dentists worldwide) supports developing evidence-based recommendations, guidelines, and regulations based on international best practices in consultation with the dental profession [22,23].

Establishing any evidence-based risk assessment method for research should go through four specific stages: (1) hazard identification, (2) dose-response, (3) risk characterization, and (4) informing decisions. Public health experts must establish a risk magnitude approach to prevent COVID-19. Using this approach can help to empower public safety in dental settings to avoid any consequences that the lack of dental care can cause [24,25].

In the 3rd International Electronic Conference on Environmental Research and Public Health—Public Health Issues in the Context of the COVID-19 Pandemic (ECERPH-3), we proposed a risk assessment approach and a COVID-19 occupational classification system. We believe that this COVID-19 risk measurement approach can be beneficial for improving the safety standards of dental care delivery. This article aims to adopt this approach for dental care delivery and reviews recommendations on safety measures for dental settings.

## 2. Materials and Methods

1. Using keywords relevant to the study (“COVID-19”, “SARS-CoV-2”, “Risk Assessment”, and “Dental Settings”), we searched to find the relevant literature published from 2019 to 2021 on two bibliographic databases (PubMed and Web of Science). Using the same period (2019–2021), we searched for guidelines and recommendations published on the official websites of several public health authorities: The Federal Service for Surveillance on Consumer Rights Protection and Human Wellbeing (Russia), the World Health Organization (WHO), the Centers for Disease Control and Prevention (US), and the British Medical Association (UK). Then, based on the search results, we analyzed the risk of contracting coronavirus (COVID-19) infection in dental settings.

2. We adopted Sechenov University Risk Assessment Criteria (RAC) and Sechenov University COVID-19 Occupational Classification System that we suggested earlier (Mitrokhin et al., 2021) in the 3rd International Electronic Conference on Environmental Research and Public Health—Public Health Issues in the Context of the COVID-19 Pandemic (ECERPH-3) to develop an approach for measuring the risk of contracting COVID-19 infection in dental settings.

3. Based on the Sechenov University Risk Assessment Criteria (RAC) and Sechenov University Occupational Classification System and the analyzed published literature, we recommended measures to minimize the risk of contracting COVID-19 in dental settings.

## 3. Results and Discussion

It is possible to assess risk magnitude of contracting the novel coronavirus (COVID-19) infection through using the following sanitary and hygienic risk assessment criteria (RAC): infectious dose (D), number of contacts (n), time (T), distance from the infectious source (H), and shielding (M) [26,27,28,29].

The risk magnitude of contracting COVID-19 infection can be measured using the following measurement scale: High Risk (HR), Medium Risk (MR), Low Risk (LR). A novel risk assessment method (Table 1), together with a novel occupational classification system (Table 2) were developed earlier at the 3rd International Electronic Conference on Environmental Research and Public Health (Mitrokhin et al., 2021), and as we believe it is possible to adapt them for measure the risk magnitude of the coronavirus (COVID-19) infection in dental settings.

Sechenov University COVID-19 Risk Assessment Approach (Based on the RAC):

- High Risk (HR) = *D* (1 + *n*) × *T* × *H* × *M*; RAC for high risk (HR) of COVID-19 infection: many contacts with infected people (more than one contact), the distance from an infected person is less than 1 m, the contact time is more than 15 min, and the lack of personal protective equipment (PPE).
- Low Risk (LR) = *D* (1) × *T* × *H* × *M*; RAC for low risk (LR) of COVID-19 infection: a small number of contacts with infected people (not more than one contact), the distance from an infected person is more than 1 m, the contact time is less than 15 min, and the proper usage of personal protective equipment (PPE).
- Medium Risk (MR); Medium risk (MR) is when RAC does not match the Low Risk (LR) conditions: *LR* < *MR* < *HR* [26].

It is necessary to carry out several preventive measures based on the routes of transmission of COVID-19. Studies showed that its main transmission routes are respiratory droplets and indirect contact, but other vertical transmission routes have yet to be confirmed. Transmission through aerosols is not confirmed yet but should be considered for dental care workers due to the highly aerosol-generating procedures in dentistry [30,31,32].

Thus, according to the Sechenov University Risk Assessment Criteria (RAC), dental care workers are at medium to high risk of contracting COVID-19. However, implementing the Low Risk (LR) criteria in the occupational classification system can help minimize the coronavirus (COVID-19) infectious risk of dental care delivery.

Since the coronavirus pandemic began, many authors and public health authorities have suggested different risk assessment tools, and there is a difference in the assessment criteria. The World Health Organization (WHO), the British Medical Association (BMA), the United States Centers for Disease Control and Prevention (CDC), and many public health authorities and authors have suggested risk assessment approaches. They have based those frameworks and tools on expert opinion and best practice in occupational health [33].

In this study, we adopted Sechenov University Risk Assessment Approach. This risk assessment has more comprehensive criteria (risk factors): infectious dose, number of contacts, contact time, distance from an infectious source, and personal protective equipment. Assessing the risk of COVID-19 using these criteria can be more compatible for healthcare workers and managing this risk in dental settings [20].

It is possible to compare risk assessment approaches based on the criteria (risk factors) considered. For instance, among all the suggested risk assessment approaches, the CDC risk assessment involved contact time but did not cover the infectious dose. The BMA proposed a risk assessment with the following criteria: age, ethnicity, biological sex, disability, health conditions, and pregnancy. None of the BMA-suggested criteria included what we proposed. WHO suggested an approach that comprised time, distance, and personal protective equipment, but did not include the infectious dose and the number of contacts [34,35,36].

Regarding the infectious dose (D), during water irrigation for cooling of the dental or surgical sites, dental procedures generate aerosols and droplets mixed with saliva and breath that contain a significant number of pathogenic microorganisms, including bacteria or viruses. Still, there is no firm evidence that aerosols generated from dental care lead to the transmission of severe-acute-respiratory-syndrome coronavirus (SARS-CoV-2). However, many guidelines recommend observing the urgency of the epidemic situation and considering those risk factors. Usually, it is possible to explain that with a rule of thumb: the greater the imminent threat to public health, the lower the standards of evidence in early guidance. Regardless, some authors reported a possible detection of high viral loads of SARS-CoV-2 in oral fluids of patients with COVID-19 [32,37,38].

The number of patients (n) is essential to measure the infectious risk; the greater the number of contacts of dental clinic workers while performing dental procedures, the higher the risk of contamination of COVID-19, especially in the presence of airflows that may diffuse droplets to areas around dental equipment. This criterion is not limited to the number of patients in contact with the dental workers, but it includes dental practice management aspects such as limiting the number of patients in the waiting room [39].

Time (T) plays an essential role in the contamination of COVID-19, and the longer the time of both consultations and dental procedures, the higher the risk of contracting COVID-19. Most standard dental procedures such as endodontic treatment or caries removal require over 15 min [40].

Regarding the distance (H) from the infectious source, studies suggested that dental care workers can be at high risk because of proximity between dentist and patient in the operational dental setting. It is impossible to maintain the recommended safety one-to-two-meter distance [41,42,43].

Shielding (M) is an important factor for safe dental care delivery, and personal protective equipment (PPE) such as facemask, gloves, eye protection, gown, respirator are necessary to minimize the infectious risk of COVID-19 in dental settings [31].

We also suggested the Sechenov University Occupational Classification System (Table 2). This classification system classified risk according to the following factors: contact with patients, contact with infected medical instruments, ventilation, air disinfection, contact with infected surfaces, linen, and clothes, and contact with fluids such as sputum or saliva.

In the literature, there is an article that classified dental procedures in the context of COVID-19 according to biological matrices of transmission (saliva, blood, aerosol, and the timing of dental procedures). Similarly, our classification system considered contact with similar biological matrices. However, our occupational classification is more comprehensive and considered several essential parameters: air disinfection, ventilation, contact with infected instruments, and contact with infected surfaces, linen, and clothes. Regarding contact time, because of the considerable length of all procedures (over 15 min), we relied on our risk assessment tool to classify dental procedures as medium-to-high-risk procedures where the criteria of medium risk are optimal for safe dental care delivery [44].

In Sechenov University Occupational Classification System, implementing the low-risk (LR) criteria can help minimize the coronavirus (COVID-19) infectious risk of dental care delivery. This means that for providing safe dental care, it is necessary to carry out several measures: the usage of personal protective equipment while in contact with patients and their body fluids, carrying out air disinfection and ventilation of premises and disinfecting dental instruments, surfaces, linen, and clothes.

Considering the routes of transmission, environmental control of indoor air and surfaces is a fundamental strategy in limiting the spread of COVID-19 in healthcare facilities. Preserving optimal indoor microbiological quality, heating, ventilation, and air conditioning systems are essential preventive strategies [45].

According to the available literature, on surfaces made of various materials such as plastic, metal, or glass, the coronavirus can remain infectious for 2 h up to 9 days. Ideally, a high temperature such as 30 °C or 40 °C can reduce the persistence duration of highly pathogenic viruses. It is advisable to sterilize or at least disinfect instruments, textiles, and surfaces [46,47,48].

Studies proved that covering more of the body and higher-level specifications of masks and respirators could provide better protection for healthcare workers. In the literature, there is a need for more high-quality studies on the specific efficiency of personal protective equipment (PPE) in protecting healthcare workers from the risk of COVID-19. However, based on the available scientific evidence and considering the transmission routes, using PPE is necessary for minimizing the infectious risk that may include COVID-19 transmission during contact with patients and body fluids generated during dental procedures [49].

It is possible to assume that, during the COVID-19 outbreak, the measurement of risk magnitude is essential for promoting safety in dental settings (for dental care providers and dental patients). With our novel approach, it is possible to minimize the risk of contracting COVID-19 while delivering dental care. Considering this can help to realize strategies for public safety and the patients’ best interest. Besides, this approach can be beneficial for developing the outcomes of dental public health authorities.

Studies showed that because of the work environment conditions, not implementing appropriate measures of infection control can cause higher infectious risk in dental offices and among dental staff. Therefore, work safety protocols in dentistry have to be revised, and additional methods of decontamination implemented [50].

Identifying and implementing strategies and approaches to reduce the chances of infection among healthcare workers is a priority for any healthcare facility, especially during the pandemic. In addition to existing infection control standards, there is an urgent need for incorporating recent information and advancements to reduce contamination of healthcare workers, including dentists [51].

In the literature, findings showed that there are around 2600 publications on COVID-19 risk assessment and about 175 on COVID-19 risk assessment in dental settings. To date, COVID-19 represents a risk to the population and healthcare providers, including dental care workers, which indicates nothing but the importance of developing more comprehensive, competitive, and innovative risk assessment approaches.

## 4. Recommendations

### 4.1. World Health Orgonization (WHO) Recommendations for Healthcare Facilites

- The World Health Organization (WHO) published a list of existing and new recommendations to prevent SARS-CoV-2 health worker infections. This included the establishment of an infection prevention and control (IPC) program and the establishment of an occupational health and safety program. It is important to consider those recommendations and minimum requirements for safety standards in healthcare facilities [52].

### 4.2. Suggested Specific Recommendations for Dental Care Settings

- Implementing teledentistry. Teleconsultation, telediagnosis, teletriage, and telemonitoring can significantly reduce human-to-human transmission and can be useful in saving time and reducing costs [53].
- Waiting area. As COVID-19 is primarily transmitted among people through respiratory droplets, it is obligatory to schedule appointments to ensure that the number of patients in the waiting room allows an interpersonal distance of at least 1 to 2 m. The time of dental procedures should be minimized to reduce the waiting time. Moreover, practice management software can be useful to manage the waiting area [41,42,43,54].
- Sorting patients. All asymptomatic patients should be considered as people with COVID-19, and all dental procedures should be revised after positive identification of COVID-19 [55].
- Avoiding aerosol-generating procedures. Dental care workers should avoid aerosol-generating procedures to the best and prioritize the use of hand instruments such as spoon excavators in combination with chemo-mechanical caries removal agents. Additionally, if an aerosol-generating procedure needs to be performed, it should be scheduled as the last appointment of the day [56].
- Dental ergonomics. It is essential to implement a professional four-handed dentistry with the usage of regular suction. We also recommend working from the 10 or 11 o’clock position, and we recommend avoiding the 8 o’clock position as much as possible to avoid splutter [56].
- Preprocedural mouth rinse. Regardless of the limited evidence about the clinical efficacy of any mouth rinses in the reduction of SARS-CoV-2 in the dental aerosol, some literature suggested that the use of preprocedural mouth rinses has been proposed to reduce the viral load in saliva and oropharyngeal tissues, thus decreasing viral load in the dental aerosol [57].
- Rubber dam isolation. In dentistry, the usage of rubber dam (Kofferdam) can be used for isolating the operative site from the rest of the mount, and now, it has been proven that this would significantly minimize the inhalation of infectious aerosols by dental care workers [58].
- Continuous education. Dental care workers must be well-informed of the recommended practices, initiatives for attending webinars, continuing dental education programs on COVID-19, and must keep themselves updated and well-prepared with extra precautionary measures to be taken [59,60].
- COVID-19 vaccination. A large share of the world population needs to be immune to COVID-19, and there are worldwide efforts for vaccination. Unfortunately, this problem represents a challenge to public health authorities; those international efforts are not enough, and there is a problem with the availability of vaccines. In the context of COVID-19 in dental settings, it is necessary to consider vaccinating dental care workers and dental patients to maximize the safety of dental care delivery. This measure may play a significant role in the recovery of the dental industry in the post-COVID era [61].
- Other recommendations. Positioning the dental handpiece correctly, using dental unit valves, rinsing dental units, and maintaining microbiological control of water and safety of the unit users [62].

## 5. Conclusions

Dental care delivery is essential, but dentists, by nature, are always at risk of exposure to the novel coronavirus infection. The factors affecting dental care delivery include the infectious dose, number of contacts, time, distance from an infectious source, and shielding (personal protective equipment).

Assessing risk magnitude can prevent the contraction of COVID-19, and by prioritizing the epidemic situation and promoting evidence-based high-level safety standards, it is possible to administer safe dental care even in high-risk patients. In our method, we used the following risk measurement scale: high risk, medium risk, and low risk.

According to the Sechenov University Risk Assessment Criteria (RAC), dental care workers are at medium to high risk of contracting COVID-19. However, implementing the low-risk criteria in Sechenov University Occupational Classification System can help minimize the COVID-19 infectious risk of dental care delivery.

We proposed recommendations for improving safety standards in dental settings: teledentistry, safety measures for the waiting area of dental clinics, avoiding aerosol-generating procedures, applying pre-procedural mouth rinse, rubber dam isolation, continuous education, vaccination, and other recommendations.

## Figures and Tables

**Table 1:** Sechenov University COVID-19 Risk Assessment Criteria (RAC).

Risk Levels	Infectious Dose (Number of Contacts with Infected People)	Distance	Time	Shielding (Personal Protective Equipment)	Ventilation (Being in an Open or Enclosed Space)
High Risk (HR)	More than 1 contact	Less than 1 m	More than 15 min	Not using any personal protective equipment (PPE)	Being in an enclosed space without ventilation
Medium Risk (MR)	Failure to meet any of the Low Risk (LR) criteria				
Low Risk (LR)	Not more than 1 contact	1–2 m and more	Less than 15 min	Using personal protective equipment (PPE)	Being in an open space or a ventilated area

**Table 2:** Sechenov University Occupational Classification System (Based on COVID-19 Infectious Risk).

Risk Levels	Contact with Patients	Contact with Infected Medical Instruments	Contact with Infected Surfaces, Linen, and Clothes	Contact with Body Fluids Such as Sputum or Saliva	Air Disinfection	Ventilation of Premises
High Risk (HR)	Without personal protective equipment (PPE)	Without disinfection	Without disinfection	Without personal protective equipment (PPE)	Not carried out	Not carried out
Medium Risk (MR)	Failure to meet any of the Low Risk (LR) criteria					
Low Risk (LR)	With personal protective equipment (PPE)	With disinfection	With disinfection	With personal protective equipment (PPE)	Carried out	Carried out

## Data Availability

Data is available upon request.
